# Fitting soil extracellular enzyme activity into the complex network of abiotic and biotic soil properties often associated with soil health

**DOI:** 10.3389/fmicb.2025.1638267

**Published:** 2025-09-25

**Authors:** Megan G. Taggart, Douglas S. Baah, Sophie Allen, Zahoor Khan, Joerg Arnscheidt, Phil Jordan, Barry M. G. O’Hagan, Aliyu D. Ibrahim, J. R. Rao, Nigel G. Ternan

**Affiliations:** ^1^Nutrition Innovation Centre for Food and Health (NICHE), School of Biomedical Sciences, Ulster University, Coleraine, Northern Ireland; ^2^School of Geography and Environmental Sciences, Ulster University, Coleraine, Northern Ireland; ^3^Centre for Genomic Research, School of Biomedical Sciences, Ulster University, Coleraine, Northern Ireland; ^4^Soils Health Research Group, Soil Molecular and Environmental Microbiology Unit, Agri-Environment Branch, Environment and Marine Sciences Division, AFBI, Belfast, United Kingdom

**Keywords:** soil enzymology, soil health, carbon cycling, nutrient cycling, microbiome

## Abstract

In this mini review we examine how soil extracellular enzymes play a key role in nutrient cycling, but stress that their activity alone does not fully represent ecosystem processes. We emphasize the need for more contextual environmental data—such as pH, temperature, moisture and nutrient availability—for accurate interpretation of the significance of enzyme activity in carbon and nutrient (N, P) cycling in soil ecosystems. The importance of enzymes within the soil microbiome determines its inherent capacity to support crop growth and often reflects soil quality and soil health, which are in turn governed by multiple different soil properties. Soil enzymes (e.g., phosphatase, glucosidases, glycosaminidases) activity have been used as key soil health bio indicators for monitoring soil nutrient transformations in overgeneralized statements. Although soil enzymes constitute important attributes that are closely linked to the dynamics of soil nutrient transformation and make nutrients available to plants, we suggest a multi-factor assessment for soil health measurement. We propose that this can give a pulse reading of soil nutrient health at crucial times of soil, land use, and crop management practices but that care is required to incorporate temporal soil and land use properties for correct interpretation.

## 1 Introduction

Across developed countries with intensive agriculture, the majority of soils are dominated by varying proportions of grassland or arable systems, and the remainder are interspersed with peatland and forestry land cover. In all these scenarios, soil is a heterogeneous, porous, living, natural and dynamic system, which is crucial to maintaining the entire ecosystem. An increasing global population has led to the need for enhanced crop production through intensive farming in order to produce enough to ensure food security. In doing so, soils and ecosystems have become stressed known as negative plant–soil feedback (PSF) and, ultimately, develop soil sickness or fatigue (Mariotte et al., 2018). This has also led to the increased production of greenhouse gasses which are contributing to the climate crisis (Molotoks et al., 2021; Rosenzweig et al., 2020). In order to tackle these problems, further understanding of the complexities of soil health has become a major concern (Nolan et al., 2021).

Soil enzymes serve as promising indicators of soil quality due to their close link to soil biological activity, ease of measurement, and quick response to changes in soil management practices (Dick et al., 1996). Extracellular enzymes in soil are produced from microorganisms including bacteria and fungi that decompose organic matter to release useable nutrients. Measurement of soil enzyme activities has been carried out since early in the 20th century, becoming more advanced and topical by the 1990s (German et al., 2011). Due to the increased interest and research in the area, optimization of the procedures has been widely reported. However, there is disagreement around attempts to standardize both the methods used within different research groups, as well as establishing accepted criteria for the interpretation of the results (DeForest, 2009; German et al., 2011; Margenot and Wade, 2023). Optimization of the methods of soil enzyme analysis has been reviewed in detail by several groups and therefore will not be further considered in this mini-review (DeForest, 2009; German et al., 2011; Baah et al., 2025). Although, a standardized method is considered highly important for the correct interpretation of soil enzyme activities, it has still not been adopted by all research groups.

## 2 Review question

Arguably less clear from the literature is how enzyme activities can and should be correctly reported, which has led to overgeneralization and misinterpretation of the data as standard in much of the published literature (Margenot and Wade, 2023; Mori et al., 2023). A letter to the editor of “Agrosystems, Geoscience and Environment” by Margenot and Wade (2023), for example, helpfully outlines the most common errors in the interpretation of soil enzyme activities by critical review of a published work which associated multiple soil properties including crop yield with soil enzyme activity (Sainju et al., 2022). However, this mini-review outlines how the narrative of soil enzyme activity data can be improved upon by the inclusion of additional data to generate a more robust assessment of soil health. Therefore, it is proposed that careful assessment of methods should be considered for all published reports of soil enzymatic activities and a minimum requirement of methodological detail suggested and agreed upon, similar to the MIQE guidelines which outlines the minimum information required for publication of qPCR data (Bustin et al., 2009). This will also benefit researchers in publication of data where high impact journals such as “Geoderma” have specified restrictions for papers reporting on soil enzyme activities (see Guide for Authors).

## 3 Enzyme activity as an indicator of soil health

**Figure 1:**
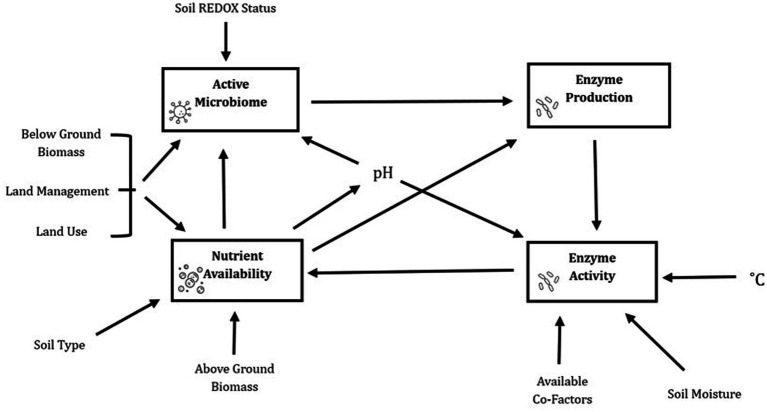
The interrelated network of factors effecting soil biogeochemical properties and processes create a complex and incomplete picture where any one unmeasured variable could drastically shift the dynamic.

It is suggested here that one such interpretation of soil enzyme activity as an indicator of “soil health” be reassessed. Soil health is considered to be the ability of the soil to function and sustain the desired ecosystem intended for a specific area in the most economical and sustainable way (Bhaduri et al., 2022). Although recognized as an important concept in soil science, “soil health” can be difficult to define in terms of measured features without an understanding of the specific requirements of a particular environment (Bhaduri et al., 2022). In this way it cannot be assumed that the same levels of soil enzymatic activity can be beneficial to the sustainability of all ecosystems. While it is generally assumed that the biological properties of soil—such as enzymatic activities—are earlier indicators of soil degradation than chemical or physical parameters (Dick et al., 1996), and because enzymes appear particularly sensitive to many land use changes, their use as bioindicators of soil quality and health has been proposed. However, enzyme activity should be reviewed primarily as a dynamic, short-term indicator of biochemical processes that contribute to, but not fully define, the longer term and more stable condition of soil health. The generality of the elusive term “soil health” encompassing so many different factors make it inappropriate to estimate using one measurable outcome (Lehmann et al., 2020; Bhaduri et al., 2022; Margenot and Wade, 2023; Mori et al., 2023). Here, we highlight the relationship between extracellular enzyme activity of soil and numerous biotic and abiotic factors as shown in Figure 1, to suggest what data should accompany soil enzyme activity measurements to report on more specific concerns.

## 4 Enzyme activity in relation to nutrient cycling

The most commonly investigated extracellular soil enzymes are *β*-1,4-Glucosidase, acid and alkaline phosphatase, β-1,4-N-acetyl-glucosaminidase and L-leucine aminopeptidase each relating to aspects of the carbon, phosphorus, and nitrogen nutrient cycles, respectively. However, when interpreting soil enzymatic activity alone and what it represents, it is heavily disputed how useful each individual enzyme activity can be in predicting the activity of the whole nutrient cycle (Margenot and Wade, 2023).

An understanding of the role of each enzyme in the context of the whole nutrient cycle is imperative to researchers to limit the overgeneralization and misinterpretation of results (Margenot and Wade, 2023). Often reported as an indicator of the whole carbon cycle, *β*-1,4-Glucosidase, primarily produced by fungi, is responsible for hydrolysis of the β-1,4-glycosidic bonds in cellulose and other related polysaccharides into various glycoconjugates which need to be further mineralized before they can be used by microbes (Zang et al., 2018). Recognizing β-1,4-Glucosidase activity as a single step in a large cycle gives merit to the requirement for additional supporting data to strengthen research findings.

**Figure 2:**
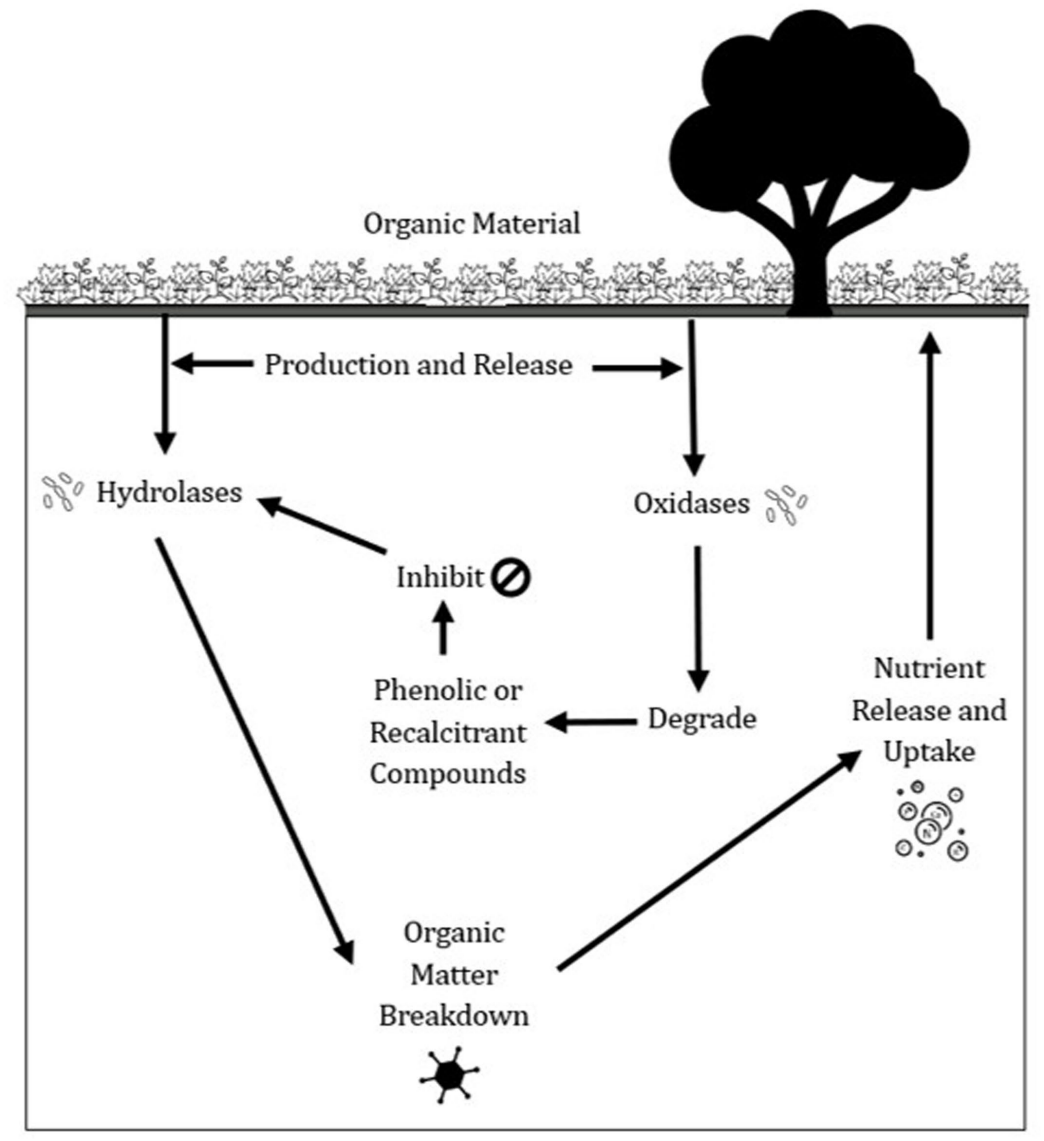
An illustration of the role of phenol oxidase (PO) and peroxidase (PPO) in soil nutrient cycling.

Other enzymes central to carbon cycling are Phenoloxidase (PO) and peroxidases (PPO), playing a crucial role in degrading recalcitrant (poly) phenolic compounds in soil organic matter (SOM) (Freeman et al., 2001). These phenolics, derived from both natural and anthropogenic sources (Balasundram et al., 2006; Michalowicz and Duda, 2007), can inhibit hydrolytic enzyme activity, thereby slowing SOM decomposition and mineralization (Figure 2). Unlike hydrolytic enzymes, PO and PPO activities have been rarely explored (Floch et al., 2007), despite their important function in overcoming phenolic inhibition by oxidatively breaking down these compounds, thus facilitating microbial access to carbon substrates (Freeman et al., 2001). However, excessive accumulation of phenolic compounds may still suppress enzyme activity via mechanisms such as pH reduction, metal chelation, and covalent bonding with amino acids, limiting nutrient availability (Min et al., 2015). Additionally, phenolics interact with physical stabilization processes, such as adsorption and aggregation, further restricting microbial metabolism (Bol et al., 2009; Kiem and Kogel-Knabner, 2002). By moderating the availability and decomposition of organic matter, PO and PPO indirectly influence soil carbon retention. Their activity plays a dual role—both enabling SOM turnover and, when phenolic concentrations are high, contributing to long-term carbon sequestration in soils (Freeman et al., 2004).

Environmental factors that highly influence the experimental measurement of extracellular enzyme activity including temperature, pH and moisture content should accompany any reporting of enzyme activity data (Yang et al., 2023). These authors propose that additional measurements of the carbon, nitrogen and phosphorus content in the soils, as a minimum, could aid in description of the activity of cycles in a soil sample. Acquiring these data in longitudinal studies would make it more valuable. Other, less commonly reported data, which could add value to such research could be measurement of gasses released from soils including carbon dioxide and methane relating to the carbon cycle.

However, the literature strongly associates enzyme activity as responsible for nutrient levels in soil, such as carbon, nitrogen and phosphorus, but it must also be understood that the available nutrient levels can also strongly influence enzyme production by microbes in a negative feedback loop that is referred to as resource allocation theory (Figure 1; Yang et al., 2023). However, it must be remembered that extracellular enzymes can become bound and stabilized within the soil matrix, becoming “abiontic” and therefore no longer associated with viable microbial cells (Nannipieri et al., 2018). Continued measurements of multiple factors will build a robust trend over time, but care must be taken to measure other factors which may influence the soil microbiome and enzyme activity such as changes in soil pH, temperature and moisture content (Yang et al., 2023). Other information including land use and management including crop cover, tillage, grazing and fertilizer use would also be helpful data to include due to the potential effects on soil enzyme activity (Yang et al., 2023).

## 5 Association of enzyme activity to microbial populations

The soil microbiome refers to the microorganisms in the soil including bacteria, fungi and protists, and somewhat controversially, viruses. As the main producers of extracellular enzymes, it is reasonable to place importance on the soil microbiome in enzyme analysis, but care must be taken not to oversimplify the connection. It has been summarized that the confusion around the factors effecting the composition of the soil microbiota is due to there being no one biotic or abiotic determinant that is consistently the most important in all environments (Fierer, 2017). This could equally apply to a multitude of environmental concepts including soil extracellular enzyme activity.

It has been suggested that the composition of microbial communities within soil can be used to predict the retention, release and storage of carbon within the soil due to the abundance of genes relating to enzyme production (Trivedi et al., 2016). But it should be noted that this study was limited to only soils from grain-producing regions in Australia (Trivedi et al., 2016). Other soil types, such as forest soils, peatlands, or arid soils, may exhibit vastly different microbial dynamics and enzyme activity patterns, making it difficult to generalize findings across ecosystems (Burns et al., 2013; Lladó et al., 2017). However, it has been disputed that the microbiome cannot accurately predict the rates of biogeochemical processes due to the limitations of microbiome research including bias in PCR-based analyses, the presence of relic DNA in soils which can account for up to 80% of sequenced DNA in soil (Carini et al., 2016), and even the ability to appropriately sample soil for microbiome analysis (Fierer, 2017). Reports have indicated that microbial necromass can constitute approximately 50% of the soil organic matter pool, with living biomass less than 5%, and only a small proportion of that being active at a time (Sokol et al., 2022).

Although it is understood that microbiome research has limitations, it still provides valuable insights provided that the DNA extraction and sequencing methods used are not particularly suited to one type of microorganism (Garg et al., 2024). An alternative method using stable isotope probing linked with metagenomics has been able to identify active microbial populations through incorporation of stable isotopes into newly synthesized nucleic acids (Vyshenska et al., 2023). However, this technically intensive approach may be too specialized for routine testing.

Again, research over multiple timepoints or changing conditions has proved most insightful where in prolonged drought conditions, the ratio of copiotrophic to oligotrophic prokaryotes in the soil microbiome of forests has continuously changed with a notable increase in oligotrophic phyla over time and an increase in organic carbon (Jaeger et al., 2024). Although this cannot be directly deemed responsible, additional measurements of soil parameters in this study has given a strong evidence base for their arguments (Jaeger et al., 2024). It is inferred *a priori* that the soil carbon content increase was due to the reduction in prokaryotic copiotrophic phyla to primarily metabolize it.

However, the cyclic nature of environmental processes would reason that upon increased soil carbon content, eventually there will be a rise in copiotrophic phyla to equilibrate the ecosystem as is seen in areas of forest fire where copiotropic phyla are in increased abundance (Adkins et al., 2022). This is where extracellular enzyme data could be added to indicate the metabolic processes within the soil, providing a clearer, more robust picture.

What cannot be directly inferred by extracellular enzyme activity data is nutrient limitation for two reasons. First being the presence of abiontic enzymes in soil which have been stabilized in the soil matrix and are no longer associated with viable microbial cells. These enzymes can remain catalytically active for extended periods and do not necessarily reflect current microbial metabolic demand (Nannipieri et al., 2018). Although the exact proportion of abiontic to total extracellular enzymes can vary across soil types and conditions, however, they represent a non-negligible fraction of measured extracellular enzyme activity, especially in older or organic-rich soils (Allison, 2006; Burns et al., 2013).

Second, microbial responses to nutrient limitation are complicated by the co-existence of diverse taxa within the same soil microenvironment. Different microbial groups may experience distinct nutrient constraints and regulate enzyme production accordingly (Rosinger et al., 2019; Fan et al., 2021). When facing multiple nutrient limitations, microbes do not necessarily prioritize one nutrient over another in a linear fashion. Instead, enzyme production is often shaped by stoichiometric demands (e.g., C: N: P ratios) and energy optimization strategies (Abay et al., 2023) For instance, microbial communities may invest in a suite of extracellular enzymes targeting multiple substrates simultaneously, reflecting co-limitation or internal regulatory trade-offs. Recent global analyses confirm that microbial enzyme allocation commonly reflects simultaneous limitations by N and P, especially in tropical and temperate soils, supporting the concept of ecoenzymatic stoichiometry as a key control of nutrient acquisition (Tian et al., 2023). This complexity challenges the assumption that a dominant extracellular enzyme activity signal equates to a single limiting nutrient, particularly in systems with high spatial heterogeneity or functional redundancy among microbes.

## 6 Is RNAseq a worthwhile approach?

Multiple research groups have suggested that gene expression techniques could be used to measure enzyme activity within the soil with greater specificity. While meta transcriptomics is promising, extracellular enzyme activity is regulated not only by intracellular gene expression but also by a range of abiotic factors including soil pH, composition, temperature, and moisture, all of which strongly influence enzyme stability and turnover (Burns et al., 2013; German et al., 2011; Yang et al., 2021; Yang et al., 2023). As a result, gene expression levels do not always correlate directly with measured enzymatic activity (Nannipieri et al., 2018). Moreover, high gene abundance or expression does not necessarily guarantee enzyme production, as it can be influenced by post-transcriptional regulation, environmental stress, or energy constraints. Nevertheless, gene expression measurements could still provide valuable insight into microbial response strategies by indicating how microbes react to environmental stimuli, regardless of protein expression. Therefore, RNAseq should be seen not as a proxy of enzyme activity but rather as a complementary tool that offers upstream information about microbial functional potential.

A more effective use of RNAseq in soil studies could be to identify the soil conditions which drive changes in microbial gene expression particularly those relating to nutrient cycling. Rather than attempting to predict the “next step” in the biochemical cycle (Figure 1), measurement of the soil properties may be better used to work backword and infer the underlying causes of observed changes. A thorough assessment of nutrient availability to the microbes, as well as measurement of other environmental factors affecting enzyme activity such as pH, temperature and moisture content is required alongside RNAseq data to develop a comprehensive understanding of soil dynamics.

## 7 Conclusion

Conclusively, measurement of enzyme activity in soils provides only a snapshot of the activity of a specific enzyme at the time of sampling, under the conditions in which the measurement was taken. However, in combination with other data such as the soil carbon and nitrogen content, microbial community analysis along with records of land use and management can generate a clearer picture of “soil health.” The complex network of soil properties and their interactions dynamically shift, and therefore the more measurements that can be made to any one soil sample will aid in describing the soil environment. However, it must be noted that one singular measured property must not be definitively assigned as solely responsible for any individual soil property or used as a sole indicator of soil health due to the complexity of soil interactions. Depending on the aspects of soils health that researchers aim to investigate, a number of specified measurements should be taken with careful attention to avoid interpreting corelation as causation.
